# Treatment-associated *TP53* DNA-binding domain missense mutations in the pathogenesis of secondary gliosarcoma

**DOI:** 10.18632/oncotarget.23517

**Published:** 2017-12-20

**Authors:** Margaret Pain, Huaien Wang, Eunjee Lee, Maya Strahl, Wissam Hamou, Robert Sebra, Jun Zhu, Raymund L. Yong

**Affiliations:** ^1^ Departments of Neurosurgery and Oncological Sciences, Icahn School of Medicine at Mount Sinai, New York, NY, USA; ^2^ Department of Genetics and Genomic Sciences, Icahn School of Medicine at Mount Sinai, New York, NY, USA

**Keywords:** glioblastoma, gliosarcoma, TP53 mutation, temozolomide, mutagenesis

## Abstract

**Background:**

Gliosarcoma is a rare variant of glioblastoma (GBM) that exhibits frequent mutations in *TP53* and can develop in a secondary fashion after chemoradiation of a primary GBM. Whether temozolomide (TMZ)-induced mutagenesis of the *TP53* DNA-binding domain (DBD) can drive the pathogenesis of gliosarcoma is unclear.

**Methods:**

We identified a case of a primary GBM that rapidly progressed into secondary gliosarcoma shortly after chemoradiation was initiated. Bulk tumor was collected and gliomasphere cultures derived from both the pre- and post-treatment tumors. We performed targeted DNA sequencing and transcriptome analyses of the specimens to understand their phylogenetic relationship and identify differentially expressed gene pathways. Gliomaspheres from the primary GBM were treated with TMZ and then analyzed to compare patterns of mutagenesis *in vivo* and *ex vivo*.

**Results:**

The pre- and post-treatment tumors shared *EGFR*, *CDKN2A*, and *PTEN* mutations, but only the secondary gliosarcoma exhibited *TP53* DBD missense mutations. Two mutations, R110C, and R175H, were identified, each in distinct clones. Both were base transitions characteristic of TMZ mutagenesis. Gene expression analysis identified increased JAK-STAT signaling in the gliosarcoma, together with reduced expression of microRNAs known to regulate epithelial-mesenchymal transition. *Ex vivo* treatment of the GBM spheres with TMZ generated numerous variants in cancer driver genes, including *TP53* and *CDH1*, which were mutated in the post-treatment tumor.

**Conclusions:**

TMZ-induced *TP53* gain-of-function mutations can have a driving role in secondary gliosarcoma pathogenesis. Analysis of variants identified in *ex vivo* TMZ-treated gliomaspheres may have utility in predicting GBM evolutionary trajectories *in vivo* during standard chemoradiation.

## INTRODUCTION

A rare but well-recognized variant, gliosarcoma comprises approximately 2% of all cases of glioblastoma (GBM) and has a poorer prognosis [1, 2]. It is characterized by a biphasic appearance of mesenchymal or rhabdoid components on a background of the poorly differentiated astrocytic cells classically observed in GBM. Secondary gliosarcoma development is usually within the context of post-treatment GBM, although development from low grade glioma has been reported. Microdissection studies provide evidence that both the sarcomatous and classical components of gliosarcoma arise from a common cell of origin, which exhibits similar genetic alterations to GBM [3, 4]. However, gliosarcoma is less frequently associated with *EGFR* amplification and rarely exhibits IDH mutations. While *TP53* mutations are more common [4–7], varying characterization methods and under-sampling make it difficult to compare reported mutation rates with larger cohorts such as TCGA, which contains few gliosarcoma samples.

Given their usual occurrence outside the context of *IDH1/2* mutations, the oncogenic role of *TP53* mutations in gliosarcoma and primary GBM may be different than in IDH-mutant lower grade glioma and secondary glioblastoma. Examination of TCGA whole exome sequencing (WES) data collected on low grade glioma and primary GBM reveals that missense mutations at codon 273 were detected in 29% of samples, compared to only 6% of p53-mutant GBM samples. Moreover, the same missense mutations were completely absent in a recent series of 25 gliosarcomas profiled by WES [6]. These differences may reflect the finding that missense mutations in various regions of the p53 DNA-binding domain (DBD) have different effects. For example, R248 and R273 make contact with DNA targets, while R175, G245, R249 and R282 control the conformation and therefore specificity of the DNA-binding surface [8].

In epithelial cancers, *TP53* mutations have been linked to the epithelial-to-mesenchymal transition (EMT), which is associated with acquisition of spindle cell morphology, vimentin expression, and nuclear expression of TWIST1 and SNAI2 [9]. Many of these changes have been observed in the sarcomatous component of gliosarcomas, suggesting that EMT in carcinomas and sarcomatoid change in GBM may share common mechanisms. *TP53* DBD mutations, in particular those affecting R175 and R248, have been implicated in EMT. These missense mutations appear not only to abrogate the tumor suppressor functions of wildtype p53, but also initiate aberrant binding with non-traditional transcription factors, oncoproteins, and gene regulatory regions important in EMT [10–12].

Here, in a detailed comparative analysis of bulk DNA and gliomaspheres isolated from an IDH-wildtype GBM and its post-treatment recurrence as a secondary gliosarcoma, we identify two p53 DBD missense mutations as drivers of sarcomatoid change in GBM. Leveraging matched pre- and post-treatment derived gliomasphere cultures, we also explored whether an analysis of temozomide-induced variants, *ex vivo*, would provide useful predictive information about the evolutionary *in vivo* trajectory exhibited by the tumor.

## RESULTS

### Immunohistochemical characterization

**Figure 1:**
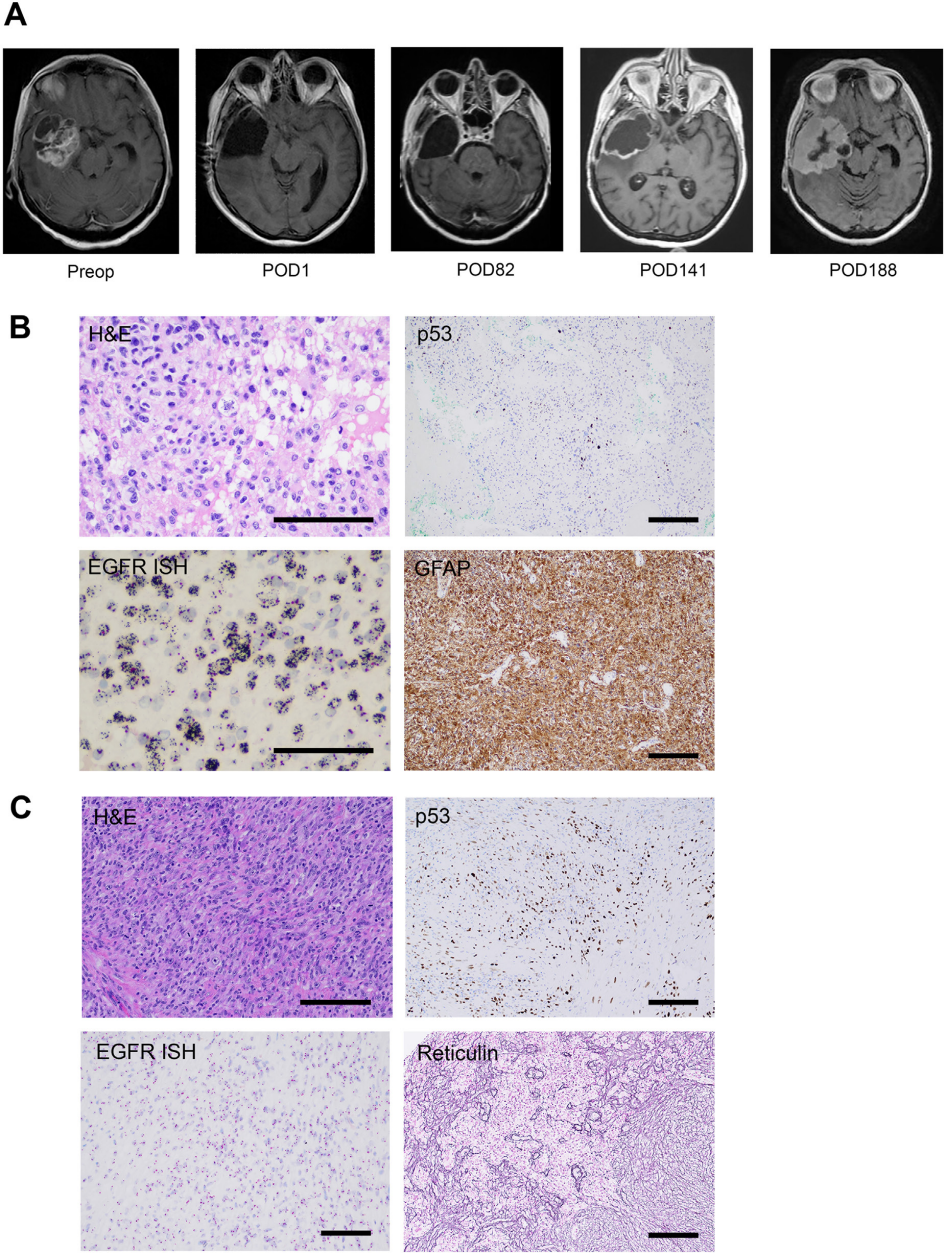
Radiographic and immunohistochemical characterization of GBM1 and SGS1 **(A)** Preoperative and serial postoperative axial T1 contrast-enhanced brain MRIs. **(B)** Stains of GBM1 demonstrating astrocytic morphology of tumor cells on H&E, p53 positive staining of 5% of tumor cells, amplification of *EGFR* on *in situ* hybridization (ISH), and positive staining for the astrocytic marker GFAP. **(C)** Stains of SGS1 demonstrating spindle cell morphology on H&E, p53 positive staining of 30% of tumor cells, lack of *EGFR* amplification on ISH, and positive reticulin staining of sarcoma-like tumor regions. Scale bars 100μm.

A 57-year-old female patient was diagnosed with a 5 × 6 cm right temporal primary glioblastoma and underwent gross total resection (Figure 1A). Histopathological analysis of the primary tumor (GBM1) demonstrated features of necrosis and microvascular proliferation consistent with glioblastoma. Staining for IDH1 R132H was negative and nuclear ATRX was intact. The Mib-1 proliferation index was 40%. p53 staining was positive in 5% of tumor cells. EGFR CISH staining revealed >20 copies of the gene per tumor cell (Figure 1B). MGMT promoter methylation was negative by pyrosequencing, and 1p19q codeletion was not detected on FISH.

Following a gross total resection of her primary tumor, the patient was treated with the Stupp regimen and remained clinically stable for 20 weeks. She completed cycle 1 of adjuvant TMZ on post-operative day (POD) 98 and cycle 2 on POD 130. At the 20-week follow-up MRI, mildly increased smooth enhancement of the periphery of the surgical cavity was noted, which was associated with slightly increased perfusion and permeability parameters, but no enhancing nodularity (Figure 1A). Over the subsequent 6 weeks, the patient experienced a decline in her neurological condition. Repeat imaging demonstrated massive expansion of the thin rim of enhancement, filling in the surgical cavity. She underwent re-resection via right temporal craniotomy and recovered well.

In contrast to the primary specimen, the recurrent tumor (SGS1) demonstrated a biphasic appearance of regions predominated by spindle cells with eosinophilic cytoplasm in a collagenous stroma, and other regions harboring the previously identified features of GBM (Figure 1C). Tumor cells demonstrated patchy positivity for GFAP with strong reticulin staining in GFAP negative areas. The Mib-1 index was 40%. p53 staining was positive in 30% of tumor cells. EGFR CISH staining indicated the absence of amplification. MGMT promoter methylation was again negative by pyrosequencing.

### Phylogenetic analysis of bulk tumor and derived gliomaspheres

**Figure 2:**
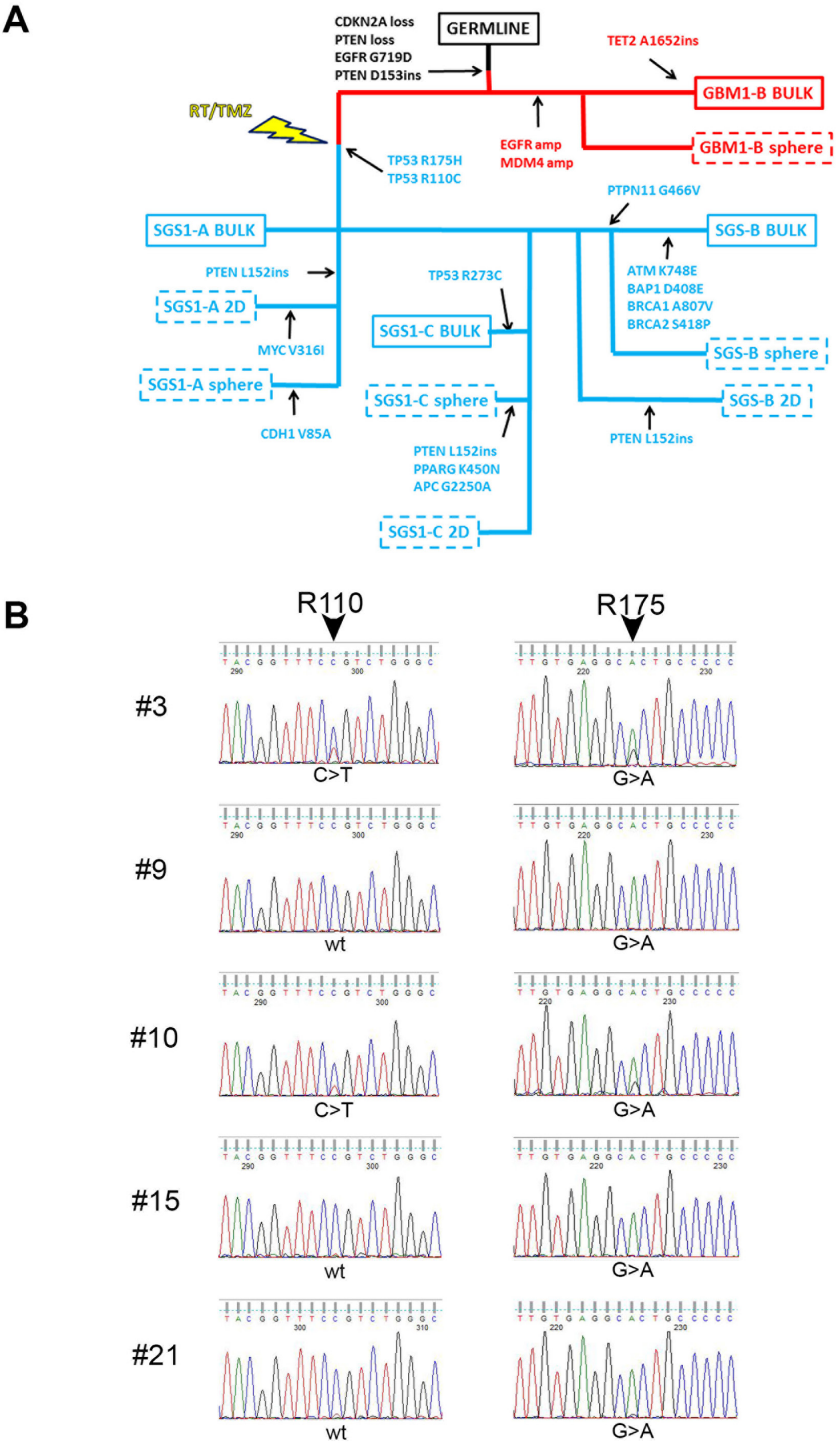
Targeted DNA sequencing reveals relationship between GBM1 and SGS1 **(A)** SNVs common and private to each of the bulk specimens and derived cultures obtained from GBM1 and SGS1 were used to construct a phylogenetic tree. GBM1 specimens are denoted in red and SGS1 specimens are denoted in blue. Dashed boxes indicate cultured specimens from bulk tissues, which are denoted by solid boxes. **(B)** Sanger chromatograms of five single cell-derived colonies from SGS1 sector B sequenced at *TP53* exons 4 (left) and 5 (right). Colonies 9, 15 and 21 exhibit the R175H (CGC>CAC) but not the R110C (CCG>CTG) mutation.

To elucidate the phylogenetic relationship between GBM1 and SGS1, we undertook Ion PGM targeted DNA sequencing of a panel of 150 genes (Oncomine Comprehensive Panel v2) known to contain mutational hotspots prevalent in the most common cancers. We analyzed the patient's germline DNA; bulk primary tumor in two sectors and corresponding derived gliomaspheres (sector A bulk tissue contained <10% tumor cells and did not produce gliomaspheres); and bulk recurrent tumor in three sectors and corresponding derived gliomaspheres and attached cultures grown in serum-containing medium (2D). By considering shared and private mutations, and changes in variant allele frequencies (VAFs) between samples, a phylogenetic tree was constructed (Figure 2A). Alterations common to both tumors included *CDKN2A* loss, *PTEN* copy loss, *EGFR* G719D mutation, and a frameshift insertion in the remaining copy of *PTEN* at D153. Only the primary tumor and its derived gliomaspheres exhibited *EGFR* and *MDM4* amplification. All bulk, gliomasphere, and 2D culture DNA from all three sectors of the recurrent tumor exhibited two *TP53* missense mutations: TP53 R110C (c.328 C>T) and TP53 R175H (c.524 G>A). Whole transcriptome sequencing of all bulk and cultured specimens revealed additional variants in *MOCOS*, *CCDC77*, *ZBED6*, and *MTFMT*, and *LAMA3* that were common to all the SGS1 samples, and which are predicted to be deleterious according to functional impact score (Supplementary Figure 1 and Supplementary Table 1). Few if any of these variants, however, have been reported in other cancers, making their possible role as drivers of SGS pathogenesis less likely than the two *TP53* variants identified.

Further analysis of the VAFs of the two TP53 missense in each of the three gliomasphere cultures derived from SGS1 revealed a reciprocal relationship with the VAFs always totaling 1.00, suggesting their presence in distinct clonal populations (Table 1). We plated SGS1 sector B gliomasphere cells as single cells and performed Sanger sequencing for both mutations on each of the resulting colonies after 6 weeks of growth. We were able to isolate 3 colonies out of a total of 30 sequenced that exhibited only the R175H mutation (the remaining 27 each exhibited both mutations), confirming the existence of these two *TP53* missense mutations in distinct subclones (Figure 2B).

**Table 1:** Somatic mutations identified by targeted DNA sequencing of bulk tumor and gliomaspheres derived from GBM1 and SGS1

Gene	hg19 Coord	Base Change	Amino Acid Change	Variant Allele Frequency (Gene Copy Number)											
				GBM1-B Bulk	GBM1-B P4	GBM1-B P12	SGS1-A Bulk	SGS1-A P4	SGS1-A P17	SGS1-B Bulk	SGS1-B P4	SGS1-B P18	SGS1-C Bulk	SGS1-C P4	SGS1-C P17
TET2	4:106196617	C>CCC	Q1652fs	0.27 (2)											
EGFR	7:55241708	G>A	G719D	0.82 (24)	0.85 (16)	0.24 (2.6)	0.12 (2.6)	0.34 (2.8)	0.42 (2.8)	0.10 (2.4)	0.26 (2.3)	0.51 (3)	0.09 (2)	0.34 (2.8)	0.41 (2.8)
EGFR	7:55249028	C>G	R776G	0.04 (24)		0.22 (2.6)									
PTEN	10:89692972	T>TA	D153ins	0.45 (1)	0.72 (1)	1.00 (1)	0.23 (2)	1.00 (1)	1.00 (1)	0.21 (1.6)	1.00 (1)	1.00 (1)		1.00 (1)	1.00 (1)
PTPN11	12:112926252	G>T	G466V								0.23 (2)				
CDH1	16:68835663	T>C	V85A					0.17 (2)							
TP53	17:7577121	G>A	R273C										0.05 (2)		
TP53	17:7578406	C>T	R175H				0.23 (2)	0.68 (2)	0.52 (2)	0.23 (2)	0.80 (2)	0.75 (2)		0.67 (2)	0.70 (2)
TP53	17:7579359	G>A	R110C				0.10 (2)	0.33 (2)	0.48 (2)	0.09 (2)	0.20 (2)	0.24 (2)		0.33 (2)	0.30 (2)

Taken together, our phylogenetic analysis suggests that GBM subclones with amplification of *EGFR* and *MDM4* were eliminated by surgery and RT/TMZ treatment, while a population of tumor cells without the amplification remained viable. These surviving cells harboring *TP53* missense mutations either were present in the primary tumor in rare numbers and survived treatment, or were an ancestral clone that acquired two distinct *TP53* missense mutations as independent events in separate cells during treatment. By considering their proportions in each of the sectors of the bulk recurrent tumor and the early passage cultures (R175H:R110C ~2:1 in A and C, ~3:1 in B), we can infer that each subclone began expanding within a time interval of less than 2 cell doublings of each other, assuming constant doubling rates.

We identified additional cases of both primary (PGS1 and PGS2) and secondary gliosarcoma (SGS2 and SGS3) with a matching primary GBM (GBM2 and GBM3, respectively) from the neuropathology archives. *TP53* DBD missense mutations were identified in all specimens. Most were conformation mutations (G245S, G245V, R175H) but 1 contact mutation (R273H) and the unclassified mutations G244A, H233del, E286K, P75S and P72R were also identified. Interestingly, 3 of the 4 cases harbored two distinct TP53 mutations each, similar to our main subject. In the fourth case, PGS1, three mutations were all found at VAFs greater than 0.05 (Table 2).

**Table 2:** Key single nucleotide and copy number variants identified in additional primary and secondary gliosarcoma samples

Gene	hg19 Coord	Base Change	Amino Acid Change	Variant Allele Frequency (Gene Copy Number)						
				GBM2 FFPE	SGS2 Bulk	SGS2 P3	GBM3 FFPE	SGS3 FFPE	PGS1 FFPE	PGS2 FFPE
CDKN2A				(0)	(0)	(0)				
EGFR				(46)	(36)	(10)				
CDK4							(2)	(5)	(5.2)	
MCL1							(2)	(6.5)	(8)	
MYC									(7.3)	
PIK3CA	3:178952085	A>G	H1047R							0.16 (2)
PTEN	10:89692847	T>C	W111R	0.75 (1.4)	0.86 (1.6)	1.00 (2)				
PTEN	10:89711922	C>G	Y180ter						0.67 (2.9)	
RB1	13:48881540	A>AT	L88fs				0.20 (2)	0.42 (2)		
RB1	13:48941669	A>T	L327ter							0.13 (2.7)
RB1	13:48955538	C>T	R552ter						0.59 (2)	
TP53	17:7579472	G>C	P72R							0.53 (2)
TP53	17:7579464	G>A	P75S						0.08 (2)	
TP53	17:7578406	C>T	R175H						0.07 (2)	
TP53	17:7577584	TGGA>T	H233del	0.27 (2)	0.15 (2)	0.09 (2)				
TP53	17:7577550	C>G	G244A						0.71 (2)	
TP53	17:7577548	C>T	G245S	0.65 (2)	0.74 (2)	0.92 (2)				
TP53	17:7577548	C>A	G245V				0.18 (2)	0.32 (2)		
TP53	17:7577121	G>A	R273C							0.20 (2)
TP53	17:7577082	C>T	E286K				0.17 (2)			

### Doubling rate and TMZ sensitivity of gliomaspheres mirrors *in vivo* disease

**Figure 3:**
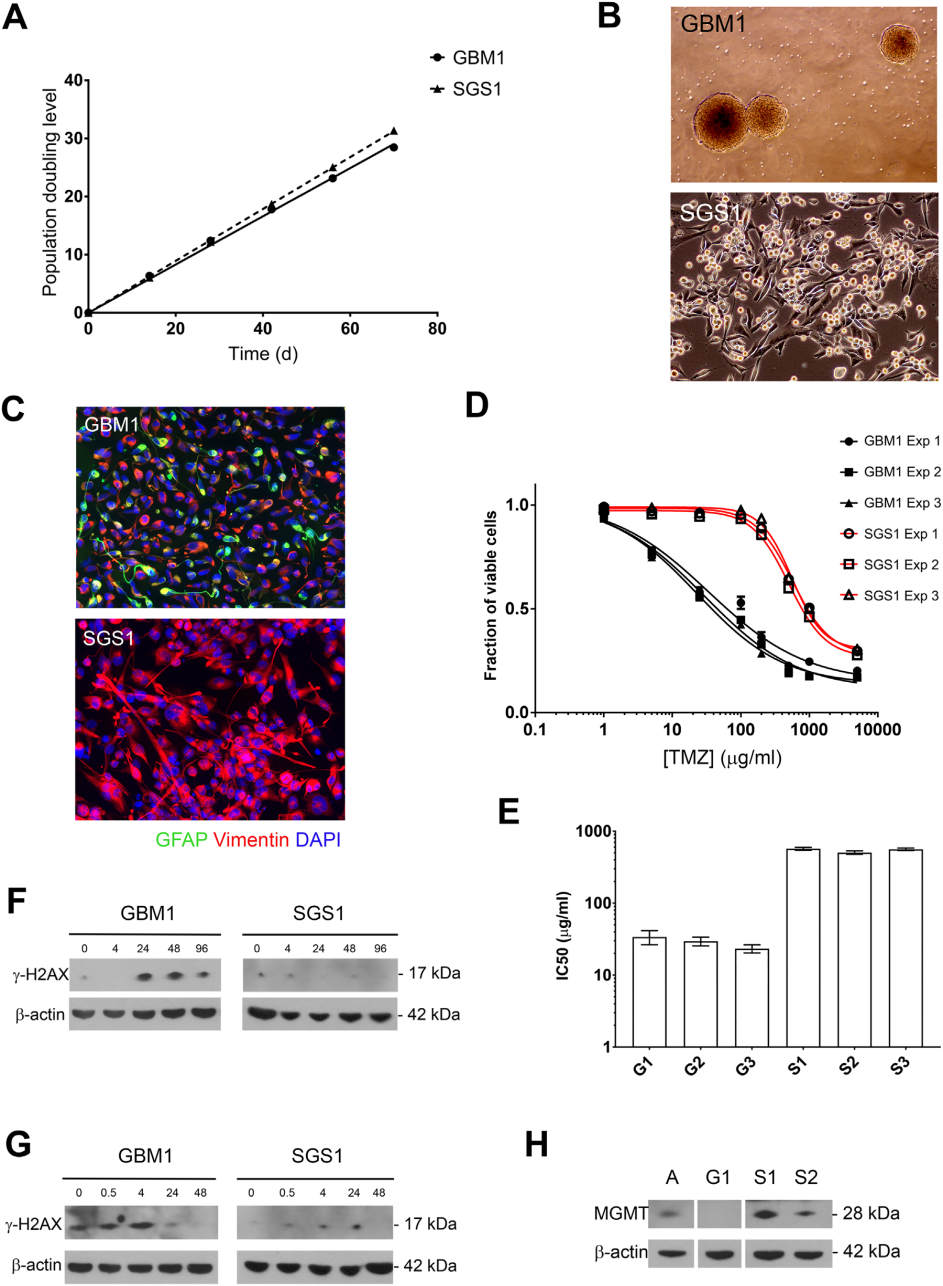
Growth rate and chemoresistance of GBM1 and SGS1 gliomaspheres **(A)** Gliomasphere cultures were serially passaged and cumulative PDL was plotted against time. **(B)** Phase contrast micrographs at 4x (top) and 10x (bottom) magnification. **(C)** Neurofilament expression by immunofluorescence (20x). Normalized cell viability **(D)** and interpolated TMZ IC50 values **(E)** from three independent experiments comparing GBM1 and SGS1 gliomaspheres. Expression of γH2AX and β-actin control in gliomaspheres by immunoblot at the indicated number of hours after exposure to TMZ 10 μg/ml for 24 hours **(F)** or 2 Gy ionizing radiation **(G)**. **(H)** Immunoblot for MGMT expression in gliomaspheres. A=cultured normal human astrocytes, G1=GBM1, S1=SGS1, S2=SGS2.

With robust gliomasphere cultures derived from both GBM1 and SGS1, we next quantified the population doubling rate of each culture over 70 days (Figure 3A). The doubling time of GBM1 cells was 2.41 days (95% CI 2.34-2.49) compared to 2.24 days (95% CI 2.22-2.26) for SGS1. Volumetric analysis of the burden of contrast-enhancing disease on POD 188 after initial resection revealed a total tumor volume of 43 cm^3^. Assuming an estimated average cell size of 2000 μm^3^ and tumor content of 50%, the numbers of R175H and R110C subclones at this time point were 6.7 × 10^9^ and 3.3 × 10^9^, respectively. This placed the time point at which the subclones began doubling between POD 114 and 118, which was between the first and second adjuvant cycles of TMZ. These calculations are consistent with the finding of minimal smooth peripheral enhancement of the surgical cavity seen on the POD 141 MRI. Our targeted sequencing panel did not detect either the R175H or R110C mutations within the primary tumor at a sequencing depth of 2000X.

Phase-contrast microscopic examination of the gliomasphere cultures derived from GBM1 and SGS1 revealed marked differences (Figure 3B). GBM1 cells grew as detached gliomaspheres in serum-free neural stem cell medium, while SGS1 cells attached to uncoated surfaces despite the absence of serum. SGS1 cells also exhibited large and irregular foot processes and tended to form sheets with multiple regions of mounding and overlapping cells. Immunofluorescence revealed increased vimentin and loss of GFAP expression in SGS1 (Figure 3C).

To determine whether the sensitivity of GBM1 and SGS1 gliomaspheres to radiation and TMZ reflected the patient's actual clinical response to these treatments, we determined the IC_50_ for TMZ for each culture (Figure 3D-3E). IC_50_ values for SGS1 were more than an order of magnitude greater than GBM1 in each of three biological replicate experiments. We then treated each culture with five days of concurrent radiation and TMZ and found that, up to 3 months after treatment, GBM1 cells exhibited no evidence of repopulation in quadruplicate T10 flasks. In stark contrast, SGS1 cells showed little signs of cytotoxicity and continued to expand. Immunoblotting for γH2AX, a marker for DNA double-strand breaks, at 0 to 96 hours after exposure to a single 5 Gy fraction of radiation or 10 μg/ml TMZ for 24 hours demonstrated that peak expression of γH2AX in SGS1 spheres was significantly less than in GBM1 spheres after either treatment, indicating resistance to double-strand break formation due to chemoradiation (Figure 3F-3G). Although both tumors did not exhibit *MGMT* promoter methylation by pyrosequencing, we nevertheless found that GBM1 spheres had a far lower level of expression than SGS1 spheres (Figure 3H). This may explain its significantly lower IC_50_ for TMZ.

Together, our results show that the R175H and R110C mutations were associated with an increase in proliferation rate that began during adjuvant TMZ; a coincident dramatic change in cellular morphology, growth pattern, and neurofilament expression; and acquired resistance to chemoradiation.

### Comparative gene expression analysis

**Figure 4:**
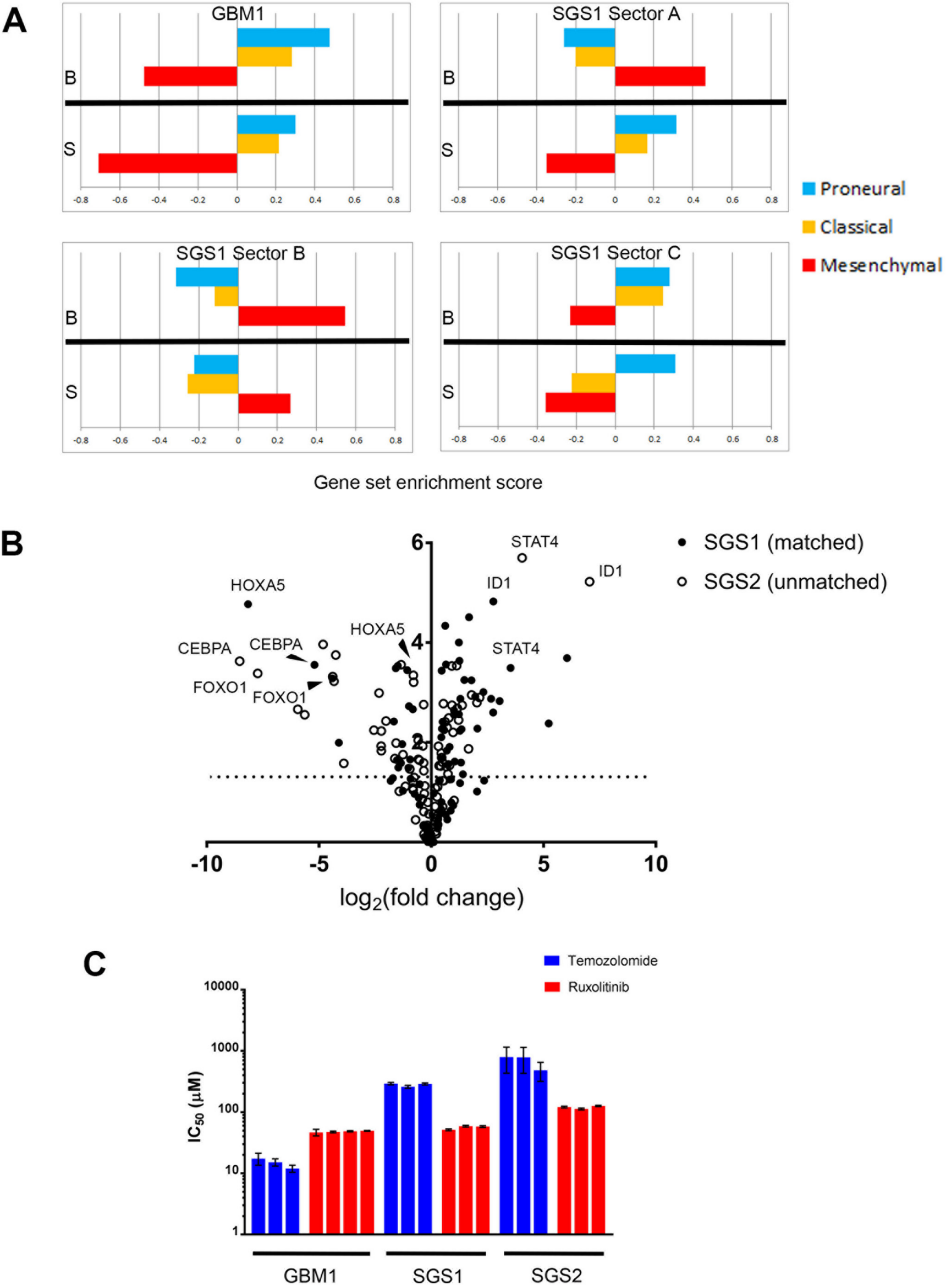
Comparative transcriptome analysis **(A)** GBM molecular subtype gene set enrichment scores on bulk tumor and gliomasphere samples. B=bulk, S=gliomasphere. **(B)** Volcano plot indicating significance and fold-change of expression level of 84 transcription factors in two gliosarcomas compared to GBM1. Y-axis is -log (p-value). **(C)** IC50 values of temozolomide and ruxolitinib for the gliomasphere lines indicated (p-value<0.0001 for interaction of drug and gliomasphere line by 2-way ANOVA). Each bar represents a biological replicate. Error bars are standard error.

To confirm that the observed mesenchymal phenotype of the recurrent tumor manifested at a transcriptomic level, we performed RNA sequencing of both bulk tumor tissue and gliomaspheres derived from GBM1 and SGS1. We used ssGSEA [13] to classify each sample into one of three GBM molecular subtypes (Figure 4A) [14]. Bulk tissue and gliomasphere RNA from GBM1 were enriched for the proneural and classical gene sets. The A and B sectors of SGS1 bulk tumor exhibited enrichment for the mesenchymal gene set, while the most medial C sector bulk expressed both proneural and classical genes and was transcriptionally more similar to GBM1 bulk tumor and gliomaspheres than SGS1 samples, possibly due to the presence of some residual GBM1 clones that did not go on to expand in culture. Gliomaspheres derived from the A and C sectors had highest enrichment scores for the proneural gene set, while B gliomaspheres were enriched for mesenchymal genes. This was associated with higher VAFs of R175H and PTPN11 G466V in the B compared to A and C cultures. Gene sets in MSigDB found to be most significantly enriched in SGS1 versus GBM1 samples included REACTOME_IMMUNE_SYSTEM, REACTOME_INTERFERON_SIGNALING, KEGG_LYSOSOME, and KEGG_CYTOKINE_CYTOKINE_RECEPTOR_INTERACTION (p<0.001, Fisher's exact test) (Table 3).

**Table 3:** Comparative gene pathway analysis of GBM1 and SGS1

Up-expressed genes in SGS1 vs. GBM1	Down-expressed genes in SGS1 vs. GBM1		
Pathway	p-value (Fisher's exact test)	Pathway	p-value (Fisher's exact test)
KEGG_LYSOSOME	1.35 x10^-5^	REACTOME_DEVELOPMENTAL_BIOLOGY	5.37 x10^-8^
KEGG_PATHWAYS_IN_CANCER	3.57 x10^-5^	REACTOME_AXON_GUIDANCE	2.54 x10^-7^
REACTOME_INNATE_IMMUNE_SYSTEM	8.11 x10^-5^	KEGG_ECM_RECEPTOR_INTERACTION	8.18 x10^-6^
KEGG_CYTOKINE_CYTOKINE_RECEPTOR_INTERACTION	1.95 x10^-4^	REACTOME_NCAM1_INTERACTIONS	1.29 x10^-4^
REACTOME_EXTRACELLULAR_MATRIX_ORGANIZATION	3.17 x10^-4^	REACTOME_EFFECTS_OF_PIP2_HYDROLYSIS	1.31 x10^-4^
BIOCARTA_CASPASE_PATHWAY	3.39 x10^-4^	REACTOME_COLLAGEN_FORMATION	1.73 x10^-4^
REACTOME_IMMUNE_SYSTEM	3.56 x10^-4^	REACTOME_INTEGRIN_CELL_SURFACE_INTERACTIONS	2.08 x10^-4^
BIOCARTA_SODD_PATHWAY	3.61 x10^-4^	KEGG_FOCAL_ADHESION	2.65 x10^-4^
KEGG_GLYCOSYLPHOSPHATIDYLINOSITOL_GPI_ANCHOR_BIOSYNTHESIS	4.74 x10^-4^	KEGG_AXON_GUIDANCE	3.23 x10^-4^
REACTOME_IL_7_SIGNALING	4.91 x10^-4^	KEGG_NOTCH_SIGNALING_PATHWAY	3.71 x10^-4^
REACTOME_POST_TRANSLATIONAL_PROTEIN_MODIFICATION	5.32 x10^-4^	PID_INTEGRIN1_PATHWAY	3.90 x10^-4^
REACTOME_POST_TRANSLATIONAL_MODIFICATION_SYNTHESIS_OF_GPI_ANCHORED_PROTEINS	5.53 x10^-4^	REACTOME_EXTRACELLULAR_MATRIX_ORGANIZATION	4.06 x10^-4^
PID_TNFPATHWAY	5.85 x10^-4^	PID_HES_HEYPATHWAY	4.16 x10^-4^
REACTOME_NUCLEOTIDE_BINDING_DOMAIN_LEUCINE_RICH_REPEAT_CONTAINING_RECEPTOR_NLR_SIGNALING_PATHWAYS	5.85 x10^-4^	REACTOME_SIGNALLING_BY_NGF	5.51 x10^-4^

We next projected differentially expressed genes from our RNAseq data onto GBM causal networks constructed from integrated gene expression, gene copy number, transcription factor target, and microRNA target data from 477 TCGA GBM samples to identify key drivers of sarcomatoid change in GBM (see Supplementary Materials). This identified upregulation of JAK-STAT signaling and the AP-1 and NF-κB1 transcription factors, as well as downregulation of microRNAs known to inhibit EMT programs (Table 4).

**Table 4:** Causal network analysis of GBM1 and SGS1

Up-expressed genes in SGS1 vs. GBM1			
Key drivers	No. of downstream nodes	Enrichment p-value	Adjusted p-value
MIR9-1-MIR9-2-MIR9-3:MIMAT0000441	36	4.45×10^-16^	8.60×10^-13^
MIR30D:MIMAT0000245	43	6.97×10^-11^	1.34×10^-7^
MA0517.1_STAT2::STAT1	22	4.76×10^-10^	9.18×10^-7^
MA0105.3_NFKB1	21	1.27×10^-9^	2.46×10^-6^
MA0099.2_JUN::FOS	133	3.21×10^-9^	6.19×10^-6^
MA0090.1_TEAD1	19	9.10×10^-9^	1.76×10^-5^
PTPN2	24	5.43×10^-8^	1.04×10^-4^
NAGA	29	9.96×10^-8^	1.92×10^-4^
MIR125A:MIMAT0000443	16	1.73×10^-7^	3.33×10^-4^
MVP	25	2.91×10^-7^	5.61×10^-4^
MA0519.1_Stat5a::Stat5b	105	3.03×10^-7^	5.84×10^-4^
MIR659:MIMAT0003337	15	4.60×10^-7^	8.88×10^-4^
MA0107.1_RELA	14	1.23×10^-6^	2.36×10^-3^
MA0473.1_ELF1	13	3.26×10^-6^	6.28×10^-3^
ARPC1B	89	7.91×10^-6^	1.52×10^-2^
**Down-expressed genes in SGS1 vs. GBM1**			
**Key drivers**	**No. of downstream nodes**	**Enrichment p-value**	**Adjusted p-value**
MIR27A:MIMAT0000084	28	7.39 x10^-13^	1.06 x10^-9^
MIR124-1-MIR124-2-MIR124-3:MIMAT0000422	22	3.18 x10^-10^	4.56 x10^-7^
MIR22:MIMAT0000077	19	6.50 x10^-9^	9.32 x10^-6^
MIR137:MIMAT0000429	18	1.77 x10^-8^	2.54 x10^-5^
MIR181B1-MIR181B2:MIMAT0000257	15	3.57 x10^-7^	5.10 x10^-4^
MIR181D:MIMAT0002821	15	3.57 x10^-7^	5.10 x10^-4^
MIR498:MIMAT0002824	15	3.57 x10^-7^	5.10 x10^-4^
MIR222:MIMAT0000279	13	2.62 x10^-6^	3.75 x10^-3^
MIR142:MIMAT0000433	12	7.10 x10^-6^	1.01 x10^-2^
MIR223:MIMAT0000280	12	7.10 x10^-6^	1.01 x10^-2^
MIR29C:MIMAT0000681	12	7.10 x10^-6^	1.01 x10^-2^
MA0162.2_EGR1	11	1.92 x10^-5^	2.74 x10^-2^
MIR24-1-MIR24-2:MIMAT0000080	11	1.92 x10^-5^	2.74 x10^-2^

As an orthogonal validation of these findings, we then used RT-qPCR arrays to compare the expression of core human transcription factors in SGS1 sector B gliomaspheres, GBM1 sector B gliomaspheres, and gliomaspheres derived from a secondary gliosarcoma resected from an unrelated individual (SGS2). This confirmed the presence of elevated STAT activity in SGS compared to primary GBM, as suggested by the RNAseq analysis (Figure 4B). We further validated this finding with assays demonstrating a significantly lower IC_50_ of the JAK2-inhibitor ruxolitinib compared to TMZ in both gliosarcoma cultures, while the IC_50_ of ruxolitinib was modestly higher than TMZ in GBM1 (Figure 4C). Significantly downregulated transcription factors common to both gliosarcomas were HOXA5, FOXO1, and CEBPA. We used oPOSSUM [15] to identify transcription factor binding sites common to the promoter regions of genes in the secondary gliosarcoma that were significantly upregulated greater than 4-fold. We found that consensus binding sites for ELK1, an ETS family transcription factor, were present in all nine upregulated genes and overrepresented compared to a background set of 24752 genes (Z-score=7.243, p=0.0047, Fisher's exact test). Another ETS transcription factor, SPIB, was also found to be enriched in the promoters of all nine genes, but at a lower significance threshold (Supplementary Materials).

Together, these results indicate that the observed phenotypic and genotypic differences between GBM1 and SGS1 correlated with an activation of gene expression networks important for EMT in carcinomas and the mesenchymal transcriptional subtype of GBM. Additionally, we detected elevated cytokine signaling via STATs and an activation of chemoresistance pathways. We found evidence that these changes may be mediated, at least in part, by ETS family transcription factors.

### Detection of TP53 mutations after *ex vivo* TMZ

Our surprising finding of the nearly simultaneous expansion of distinct *TP53* missense mutations in two separate GBM clones during treatment prompted us to ask whether this expansion could be replicated *ex vivo*. We split passage 10 gliomaspheres from GBM1 for irradiation, TMZ, or combined treatment. As a negative control, we also continued to passage untreated GBM1 gliomaspheres. Cells did not recover from combined treatment for further analysis; however, 12 weeks after treatment with TMZ alone, GBM1 cells had recovered sufficiently for NGS targeted sequencing. We detected p53 R175H and R110C at VAFs of 0.014 (28 reads, strand bias 0.6338, Phred quality score 81.2) and 0.019 (37 reads, strand bias 0.5812, Phred quality score 49.7), respectively, at a coverage depth of 1999X (Table 5). No new variants were identified in the radiated and untreated cultures compared to the pre-treatment culture. TMZ was also administered to late passage (P24) untreated GBM1 cells. Sequencing of post-treatment recovered cells revealed multiple C:G>T:A transitions in an NpCpT or NpCpC context, although the same p53 mutations were not seen. We did, however, detect the expansion of a subclone harboring a deleterious *CDH1* missense mutation (P780L/c.2339C>T, VAF 0.094 and 0.477 in a duplicate flask), a gene that was also found to be mutated in sector A cells derived from SGS1, although at a different codon.

**Table 5:** Somatic mutations identified in temozolomide-treated gliomaspheres derived from GBM1

Gene	hg19 Coord	Base Change (Context)^*^	Amino Acid Change	Variant Allele Frequency (Gene Copy Number)				
				GBM1-B P12	GBM1-B P12 IR	GBM1-B P10 TMZ	GBM1-B P24 TMZ	GBM1-B P24 TMZ flask 2
CDKN2A				(0)	(0)	(0)	(0)	(0)
MDM4				(2)	(2)	(5)	(2)	(2)
EGFR	7:55241708	G>A (GCC-)	G719D	0.24 (2.6)	0.23 (2.6)	0.33 (3)	0.25 (2.8)	0.26 (2.8)
EGFR	7:55249028	C>G (CCG+)	R776G	0.22 (2.6)	0.25 (2.6)	0.14 (3)	0.21 (2.8)	0.26 (2.8)
FGFR1	8:38282211	G>A (**TCC+**)	S251F			**0.03 (2)**		
PTCH1	9:98268751	G>A (**GCC+**)	A111V				**0.19 (2.8)**	
BRCA2	13:32893288	G>A (**TCT-**)	E48K				**0.08 (2)**	**0.29 (2)**
CDH1	16:68863600	C>T (**CCT+**)	P780L				**0.09 (1.5)**	**0.48 (1.5)**
PTEN	10:89692972	T>TA	D153fs	1.00 (1)	1.00 (1)	1.00 (1)	1.00 (1)	1.00 (1)
TP53	17:7579359	G>A (**CCG+**)	R110C			**0.02 (2)**		
TP53	17:7578406	C>T (**GCG-**)	R175H			**0.01 (2)**		
NF1	17:29667556	G>A (**GCT-**)	A2319T				**0.23 (2)**	
SMAD4	18:48604701	G>A (**GCC-**)	G508D				**0.07 (2.5)**	**0.31 (2.4)**

Putative TMZ-induced variants are indicated in bold.

^*^As per convention, context is denoted in reference to the substituted pyrimidine. + denotes that the pyrimidine substitution occurs on the transcribed strand.

*Ex vivo* treatment experiments carried out on two other MGMT promoter-methylated primary GBM cell cultures in our collection (GBM4 and GBM5) similarly detected expanded subclonal populations harboring deleterious C:G>T:A transitions in an NpCpT or NpCpC context in known cancer driver genes (Table 6). In two MGMT promoter-unmethylated primary GBM cultures (GBM6 and GBM7), no such populations were seen. These Signature 11 mutations [16] were significantly more frequent in mutations detected only after TMZ exposure versus those present prior to TMZ exposure (15/19 vs. 3/11, p = 0.0016 Fisher's exact test). In these four cases, matching recurrent GBM tissue was not available to evaluate the clinical significance of the mutations.

**Table 6:** Targeted DNA sequencing of additional MGMT-methylated and unmethylated GBMs

Gene	hg19 Coord	Base Change (Context)^*^	Amino Acid Change	Variant Allele Frequency (Gene Copy Number)							
				GBM4 P22	GBM4 P21 TMZ	GBM5 P6	GBM5 P6 IR/TMZ	GBM6 P3	GBM6 P6 TMZ	GBM7 P8	GBM7 P8 TMZ
CDKN2A						(0)	(0)	(0)	(0)	(0)	(0)
MDM4										(26)	(20)
MYCN				(112)	(74)						
PDGFRA										(5.8)	(4.6)
MSH2	2:47709922	G>A (**ACC-**)	G880D				**0.06 (2)**				
CTNNB1	3:41266078	G>A (**GCC-**)	W25ter				**0.13 (2)**				
BAP1	3:52436821	C>T (**TCC-**)	E653K		**0.13 (2)**						
BAP1	3:52436887	C>T (**TCC-**)	E631K				**0.13 (2)**				
KDR	4:55946320	G>A (**GCC+**)	P1287S		**0.08 (3)**						
PIK3R1	5:67591097	A>G (TTC-)	N564D					0.44 (2)	0.48 (2)		
APC	5:112162897	G>A (**GCA-**)	A501T		**0.11 (1)**						
APC	5:112178024	C>T (**TCC+**)	P2245S				**0.08 (2)**				
EGFR	7:55220274	C>T (GCG+)	R222C					0.20 (12)	0.03 (3.4)		
EGFR	7:55221710	C>T (CCG+)	R252C					0.05 (12)			
EGFR	7:55221822	C>T (GCC+)	A289V					0.07 (12)			
TSC1	9:135779106	G>A (**CCT+**)	L714F		**0.09 (1)**						
PTEN	10:89653807	G>C (CCA-)	M35I			1.00 (1)	1.00 (1)			(0)	(0)
PTPN11	12:112888189	G>A (TCC-)	E69K							0.05 (2)	0.06 (2)
PTPN11	12:112926900	C>A (ACA+)	T511K							0.43 (2)	0.43 (2)
TP53	17:7577524	GTGAGGATGG>G	P250del	0.48 (2)	0.47 (2)						
TP53	17:7578236	A>C (**GTA+**)	Y205D				**0.02 (2)**				
NF1	17:29556257	G>A (**ACC-**)	G875D				**0.15 (2)**				
NF1	17:29667616	G>T (TCA-)	E2339ter							0.05 (2)	0.08 (2)
NF1	17:29679366	C>T (CCG+)	R2517ter							0.05 (2)	0.07 (2)
BRCA1	17:41223252	C>T (**TCC-**)	G1560E				**0.12 (1.6)**				

Putative TMZ-induced variants are denoted in bold.

^*^As per convention, context is denoted in reference to the substituted pyrimidine. + denotes that the pyrimidine substitution occurs on the transcribed strand.

Together, these results illustrate the possibility of exploiting cultured, *ex vivo*-treated glioma stem-like cells to detect treatment-resistant subclones that may not otherwise be detected using sequenced bulk tissue samples. In our case study, these initially rare subclones went on to repopulate the tumor and appeared responsible for the sarcomatous phenotype change.

## DISCUSSION

Using detailed analyses of bulk tumor tissue and derived gliomaspheres from a case of secondary gliosarcoma, our results build a compelling case that *TP53* DBD missense mutations can drive a sarcomatous phenotype transition in GBM. We observed the appearance of two subclonal *TP53* missense mutations on a background of clonal *EGFR* missense and *PTEN* inactivating driver mutations that were shared with the matching primary GBM. Their appearance during TMZ adjuvant therapy coincided with an increase in gliomasphere proliferation rate, increased MGMT expression, a global transcriptional change towards a mesenchymal phenotype, and resistance to TMZ *in vivo* and *ex vivo*. Single-cell plating and VAF analysis of cultures derived from multiple tumor sectors demonstrated that the two mutations began to expand independently in separate GBM cells within a relatively short time interval, lending further support to the notion of their origin within a single 5-day TMZ cycle. TP53 R175H is a recognized gain-of-function mutation, while TP53 R110C has been observed in isolated cases of colorectal, squamous cell, prostate, and non-small cell lung cancer in the TCGA datasets but has not been specifically identified as a DBD conformational mutant. Our observation of the persistence of TP53 R110C in 2 of 3 bulk tumor sectors and all long-term serum-free culture suggests that it conferred a similar neoplastic fitness advantage to TP53 R175H.

This study clarifies previous work identifying a high frequency of TP53 mutations in both primary and secondary gliosarcomas [4, 5]. Most recently, Cho et al. sequenced *TP53* to a coverage depth of approximately 120x in 28 FFPE gliosarcoma specimens, including 6 with matched normal samples, and observed an overall mutation frequency of 71% [6]. Synthesizing these studies with ours, we suggest that *TP53* gain-of-function mutations are a key step in gliosarcoma pathogenesis. Although most molecular studies of gliosarcoma have reported specimens with wildtype *TP53*, an increasing sensitivity of mutation detection methods appears associated with increasing frequencies of mutation observed. Furthermore, wildtype p53 glioasarcoma exhibits an improved clinical prognosis compared to mutant p53 gliosarcoma, and may be a distinct molecular subtype despite similar histopathology [6]. Because *TP53* gain-of-function mutations are also a hallmark of IDH-mutant astrocytomas and are frequently observed in IDH-wildtype GBM without any sarcomatoid features, they may not be sufficient for gliosarcoma pathogenesis. Other necessary factors may be stromal/microenvironmental or genetic. Possible stromal factors suggested by our RNAseq GSEA include infiltrating inflammatory cells contributing to cytokine signaling, which was also observed in the study by Cho *et al*. After *TP53* SNVs, the most common genetic alterations we observed were copy number loss or inactivating SNVs of *PTEN*. Wildtype p53 is a recognized regulator of *PTEN* transcription, and PTEN acts to prevent degradation of p53 by MDM2 [17, 18]. Thus, mutations that reduce wildtype function of one gene may create a condition where acquisition of a deleterious mutation in the other is particularly advantageous.

Our study sheds new light on which wildtype p53-regulated genes must be lost for gliosarcoma pathogenesis to occur, as well as the transcription factors with which mutant p53 may be aberrantly interacting. Compared to GBM1, SGS1 exhibited significantly downregulated expression of the transcription factors CEBPA, FOXO1 and HOXA5. Examination of another secondary gliosarcoma culture in our collection, SGS2, similarly showed significant downregulation of CEBPA and FOXO1. CEBPA is a tumor suppressor that has been shown in AML to be directly activated by p53 [19]. FOXO1 and HOXA5 are not known to be directly regulated by p53, but studies in several cancer types suggest that these transcription factors may have p53-independent pro-differentiation and pro-apoptotic functions that potentiate the effects of wildtype p53 loss [20, 21]. Among the most upregulated transcription factors we identified in the secondary gliosarcoma, STAT4 is known to contain a p53 response element in its promoter [22], indicating that some wildtype binding specificities of p53 may be preserved despite the absence of the wildtype allele. Alternatively, STAT4 may be activated via other TFs such as NF-kB1 [23], which we found to be a key driver of sarcomatoid change in our causal network analysis. STAT4 is phosphorylated by JAK2, and we confirmed that the JAK2-inhibitor ruxolitinib was more cytotoxic at comparable micromolar concentrations than TMZ in two gliosarcoma cultures, meriting further exploration in preclinical models.

We identified two ETS family transcription factors, ELK1 and SPIB, as possible mediators of gene upregulation in an analysis of DNA binding site motifs. Another ETS family transcription factor, ETS2, was recently found to mediate aberrant gene upregulation by mutant p53 through a direct protein-protein interaction, resulting in etoposide resistance in breast cancer cell lines [24]. Our results are consistent with the postulate that other ETS family transcription factors may be similarly co-opted by p53 gain-of-function mutants. Binding partners of mutant p53 may have diverse effects that contribute to cancer progression, including cell cycle dysregulation [25], neoangiogenesis [26], and migration/metastasis [12]. We speculate that R175H and/or R110C interacts with ELK1 to upregulate target genes that facilitate a switch to a sarcomatoid phenotype in GBM.

The timing, relative to the patient's treatment course, of the expansion of the two TP53 mutant gliosarcoma clones suggests two possible scenarios. In the first, one or both mutations were present in a rare population of cells in the original tumor and, after treatment eliminated TMZ-sensitive p53 wildtype clones, these resistant subclones repopulated the tumor. Cho *et al*. have similarly reported the outgrowth of rare TP53-mutant subpopulations in recurrent gliosarcomas following treatment. Arguing against this in our case, however, is the fact that there was no evidence of recurrent tumor on the POD 82 MRI (approximately 36 doubling times or 6.7 × 10^10^ cells expected), unless other factors prevented proliferation of the p53-mutant subclones during that time period. Alternatively, residual ancestral clones could have acquired the TP53 mutations at approximately the same time during the first adjuvant cycle of TMZ (POD 94-98) and immediately began to proliferate. In this scenario, the patient's radiographic findings are consistent with the *in vivo* growth rate predicted by the culture.

The deleterious effects of TMZ mutagenesis in both low and high grade gliomas are well recognized [27, 28]. In SGS1, both *TP53* mutations were C:G>T:A transitions that are consistent with the so-called Signature 11 mutations [16] seen with alkylating chemotherapies. Although these transitions can also occur due to endogenous mutational processes such as spontaneous deamination of 5-methylcytosine (Signature 1), both were detected *ex vivo*, along with numerous other mutations bearing the TMZ mutagenesis signature, only after GBM1 cells were treated with TMZ. Although it seems unlikely that two distinct mutations in the same region of the same gene could be induced experimentally in this manner, we speculate that the location of the R110 and R175 codons at gene body CpG sites, which are highly methylated and thus less stable [29], increased their susceptibility to TMZ mutagenesis.

Intratumoral heterogeneity and therapy-driven evolution are major barriers to the effective treatment of GBM and other solid cancers. Our finding that *ex vivo* treatment may generate an array of subclones that mirror the actual subclones that fuel tumor recurrence *in vivo* raises the possibility that evolutionary trajectories may be anticipated to a degree. Our work is limited by the number of GBM cultures analyzed, a paucity of post-treatment recurrent GBM tissue matched to newly diagnosed GBM gliomaspheres, and use of only a single culture microenvironment in which treatment was simulated. Further work will need to be done to determine what GBM molecular subtypes may be particularly amenable to, and what culture conditions or *in vivo* models might improve the yield of, this experimental method of *ex vivo* evolutionary modeling. Because robust GBM cultures can be derived in under 1 month, and their recovery from a cycle of TMZ can occur in less than 3 months, we foresee the possibility of obtaining predictive information well in advance of the 7-month median time interval at which clinical disease progression typically occurs. This may provide the opportunity for clinicians to rationally select targeted second-line therapies, even in the absence of a biopsy specimen of the recurrent tumor.

In conclusion, this study of a striking case of secondary gliosarcoma provided a better understanding of the potential role TP53 DBD missense mutations have in activating EMT transcriptional programs and driving disease progression in GBM. Experimental treatment of cell cultures derived from the primary tumor revealed a method of modeling tumor evolution that merits further exploration. An improved ability to predict GBM evolutionary trajectories at the time of first diagnosis will facilitate the development of more rational, adaptive, and personalized therapeutic strategies.

## MATERIALS AND METHODS

### Targeted next-generation DNA sequencing

Genomic DNA extraction was conducted using Purelink Genomic DNA Min**i** kits (Thermo Fisher Scientific) to yield ample material for NGS. The Ion AmpliSeq Oncomine Comprehensive research panel version 2.0 (OCP v2.0, Thermo, #4475346) was used to characterize relevant genes at high coverage (~2000X depth) for variant characterization purposes using a pool of multiplexed primers that amplify the targeted loci of frequent mutation in known oncogenes and tumor suppressor genes. After amplification, the libraries were prepared with the Ion AmpliSeq Library Kit 2.0 (Thermo, #4475345) using Ion Xpress Barcode Adapters 1-96 Kit (Thermo, #4474517) to barcode each single cell, group of pooled cells or bulk tumor, following standard manufacturer's instructions. Briefly, 30 ng of each sample were amplified using the Ion Torrent OCP v2.0 panel and specified Ampliseq cycling conditions for the pool. Following PCR amplification, the primers were partially digested with the proprietary FuPa enzyme and each sample was barcoded with a unique IonExpress barcode (1-96). Finally, a 1.5X bead purification was performed with Agencourt AMPure XP Reagent (Beckman Coulter, #A63880) following the instructions in the Ion Ampliseq Library Prep protocol to clean up the sample and remove adapter dimers. All samples were quantified with the Ion Library TaqMan Quantitation Kit (Thermo, #4468802) using a 1:100 dilution of each library in a 10 μL reaction volume, with 2 technical replicates per sample. Following quantification, all samples were individually normalized to 100 pMol and 2 μl of each normalized library were combined to form a pool of 96 uniquely barcoded samples at a final concentration of 100 pMol. 25 μl of this pool was used for priming the sequencing chip. Ion Torrent chip priming and sequencing were carried out using the Ion Torrent S5XL system and the Ion Chef instrument with reagents from the Ion 540 Kit-Chef (Thermo, #A27759). Briefly, the Chef was used to bind each library DNA fragment to Ion Sphere Particles (ISPs) and clonally amplify each fragment by emulsion PCR. Amplified DNA fragments were then bound to streptavidin-coated beads and template negative ISPs were washed away. Template-bound ISPs were then prepared for sequencing by loading onto one Ion Torrent S5 540 chip for sequencing on the Ion S5XL sequencing system. The primed 540 chip was sequenced on the Ion Torrent S5XL System with library read length set at 200 bp and 520 flows per chip, with all other instrument settings set to the manufacturer's default for the Ion 540 Kit. Analyses of sequencing raw data were performed with Ion Torrent Suite (version 5.0.2) using the “coverageanalysis” and “variantcaller” plugins (with somatic/low stringency settings for the “variantcaller”), with all other settings for the run report set to the manufacturer's default.

### Establishment of gliomasphere cultures

Freshly resected tumor tissue was washed and digested as previously described [30]. After centrifugation, cells were resuspended in 5 ml NeuroCult NS-A Proliferation Medium (Stemcell) supplemented with recombinant human EGF (20 ng/ml), bFGF (10 ng/ml), and 0.0002% heparin, then cultured inT25 tissue culture flasks. The medium was changed at 48 hours and then every 3-4 days. Change in population doubling level was calculated using: ΔPDL = log_2_(cells harvested/cells seeded).

### Phylogenetic analysis of samples

The “variantcaller” output on each bulk and cultured sample was filtered to exclude variants occurring at a frequency of less than 0.05. These were further filtered to exclude identical variants occurring in the matched germline DNA sample, as well as non-coding or synonymous coding variants. The remaining variants were then sorted by number of samples affected in descending order. Variants that were detected in all samples were placed on the trunk and branching points were added to divide samples into progressively smaller groupings until each sample appeared on an individual branch. Reconfigurations of the tree, not affecting the underlying topology, were performed to group samples from the same bulk sectors together where possible. RNA-seq data were analyzed using the GATK best practices workflow (https://software.broadinstitute.org/gatk/documentation/article.php?id=3891) to call SNVs. SNVs that differed between GBM1 and SGS1 samples at a significance threshold of p<0.02 (Fisher's exact test) were then used to cluster the samples hierarchically in an unsupervised fashion.

### Time of clonal expansion calculation

Let *c* be the doubling time of R175H and R110C subclones (we assume they are similar because their proportions remained relatively fixed over multiple passages in each culture). If *t* is the amount of time R110C has been doubling, and *t* + *x* is the amount of time R175H has been doubling, then the ratio of R175H cell numbers to R110C cell numbers at any given time can be expressed as:

$$\frac{2^{\frac{t+x}{c}}}{2^{\frac{t}{c}}}$$

Experimentally, we observed that this ratio was less than 4:1 in all cultures, so:

$$\frac{2^{\frac{t+x}{c}}}{2^{\frac{t}{c}}}<4$$

Solving for *x*, the time interval between when the subclones started to double:

$$2^{\frac{x}{c}}<4$$

$$\frac{x}{c}<2$$

x<2c

Therefore, the subclones began doubling within 2 doubling times of each other.

### IC_50_ assays

Gliomaspheres were digested into single cells with Accutase and seeded in laminin-coated 96-well plates with serum-free medium. Plates were incubated at 37°C, 5% CO_2_, and 90% humidity until 50% confluence. The medium was then replaced with fresh medium containing temozolomide at various concentrations (0-5000 μg/ml). Six technical replicates were performed for each concentration. After incubation for 66 hours, 20 ml of MTS reagent (Promega) was added and incubated for an additional 4 hours. Plates were read at absorbance 490 nm.

### Immunohistochemistry and immunofluorescence

IHC analysis of bulk tumor specimens was performed on formalin-fixed, paraffin-embedded slides in a CLIA-approved pathology laboratory. Immunofluorescence of gliomasphere cells was performed as previously described [30]. Primary antibodies were: Sox2 (Cell Signaling, cat# 3579, dilution 1:400); Nestin (Santa Cruz, cat# sc-23927, dilution 1:200); MAP2 (Cell Signaling, cat# 4542, dilution 1:50); GFAP (Cell Signaling, cat# 3670, dilution 1:300); vimentin ( Cell Signaling, cat# 5741, dilution 1:100).

### Western blot

Protein was extracted from cells and immunoblots performed as previously described [30]. Primary antibodies were: β-actin (Cell Signaling, cat# 3700, dilution 1:2000); MGMT (Cell Signaling, cat# 2739, dilution 1:1000); γH2AX (Cell Signaling, cat#9718, dilution 1:1000).

### RNA sequencing and gene expression analysis

Total RNA was isolated from cells and tumor tissue using RNeasy Mini kits (Qiagen) and provided to the Mount Sinai Genomics Core Facility for 100nt single-read sequencing on the Illumina HiSeq 2500 instrument to generate on ~42 million reads per sample. DEseq2 [31] was used to identify differentially expressed genes between GBM1 and SGS1 samples at an adjusted p-value of 0.001 (Fisher's exact test). Identification of enriched pathways using Fisher's exact test was performed as previously described [32]. Single sample GSEA was performed using the GSVA package in R with the options maseq=TRUE and mx.diff=FALSE [13]. Gene set signatures were obtained from Wang *et al* [14].

### Construction of GBM-specific regulatory network and key driver identification

We *de novo* constructed a GBM regulatory network using a previously described procedure [33, 34] from 477 TCGA GBM tumor samples [35] by integrating gene expression and DNA copy number variation data [36, 37]. We further incorporated the causal connectivity between miRNA targets and transcription factor (TF) targets into a network model as scale-free priors (see details in following section). Based on the causal network constructed, we performed a key driver analysis (KDA) [37] to identify regulators for the gene sets. The KDA takes as input a set of genes and a gene causal (directed) network. The subnetwork of a set of genes was identified by searching their neighboring genes, and key drivers were identified by considering number of downstream nodes.

### Determination of TF-target connectivity

To determine TF-target connectivity, we inferred sample-specific TF activity [38, 39] and miRNA activity [40] based on gene expression profiles and miRNA expression profiles of 477 TCGA GBM tumor samples [35]. We downloaded 205 position-specific weight matrices (PWMs) that represent individual transcription factors (TFs) from the JASPAR CORE database [41], and obtained the genome sequence for *Homo sapiens* from the R Bioconductor package *BSgenome.Hsapiens.UCSC.hg19*. We further considered tissue-specific accessible DNA for TF binding. Specifically, we used DNase I hypersensitivity regions of two available glioblastoma cell lines A172 and H54 as the cis-regulatory sequence of each gene [42]. The TF binding affinities were estimated based on the PWM of TFs and cis-regulatory sequences. For each sample, linear regression of the genome-wide mRNA expression on the total promoter affinity for each TF was performed. The regression coefficients of the total promoter affinity were interpreted as sample-specific TF activities. We performed this procedure with different sizes of cis-regulatory sequence, 1 kb through 10 kb of upstream and downstream sequences, then selected optimal sizes of cis-regulatory sequence by enrichment between genes with high total binding affinity and genes whose expression level are correlated with TF activities. The inferred TF activity with the selected optimal sizes of cis-regulatory sequence was used to determine its functional target genes that are defined as the genes with high total binding affinity by that TF and significant expression correlation with the inferred TF activity.

### Determination of miRNA-target connectivity

To determine miRNA-target connectivity, we inferred miRNA activity based on expression levels of miRNAs and their predicted target genes in a previous study [40]. We used a collection of predicted target genes for 1537 unique mature miRNAs from TARGETSCAN (www.targetscan.org) that considers all conserved miRNA binding sites inherited from 23-way alignments of UTR sequences [43]. In order to obtain robust results, we filtered out miRNAs whose number of target genes are smaller than 100. Among these miRNAs, we further focused on miRNAs whose predicted target genes’ expression levels and their own expression levels are available, resulting in 149 miRNAs. The causal connectivity between miRNA and target was defined by the high correlation between miRNA activity and expression levels of predicted targets from TARGETSCAN.

### RT-qPCR arrays

Total RNA (0.5 μg) was used to synthesize cDNA using the RT^2^ First Strand kit (Qiagen). This cDNA was added to the RT^2^ SYBR Green Mastermix, applied to the Human Transcription Factors RT^2^ Profiler PCR Array (Qiagen, cat# PAHS-075Z), and amplified in a StepOnePlus cycler (Applied Biosystems) using the manufacturer's protocol.

### *Ex vivo* treatment of gliomasphere cultures

For radiation treatment, cells cultured in serum-free medium were placed in T25 flasks in an X-ray irradiator and exposed to either a single 5 Gy dose (for γH2AX response experiment) or daily 2 Gy fractions for 5 days (for subsequent IonPGM targeted sequencing). For TMZ treatment, cells were resuspended in serum-free medium containing TMZ 10 μg/ml (0.05 mM) daily for 5 days. Once re-formation and enlargement of gliomaspheres occurred, cultures were passaged every 3-7 days.

### Ethics statement

Patients providing germline DNA and fresh tumor tissue for sequencing and culture were consented and registered in the IRB-approved Mount Sinai Cancer Biorepository protocol. Archival specimens without matching germline DNA were obtained in a deidentified fashion from the Department of Pathology under the Code of Federal Regulations exemption 45 CFR 46.101(b)(4).

## SUPPLEMENTARY MATERIALS FIGURE AND TABLE


