# Supplementary material for: Treatment-associated TP53 DNA-binding domain missense mutations in the pathogenesis of secondary gliosarcoma
Source: Oncotarget. 2017 Dec 20;9(2):2603–21. doi: 10.18632/oncotarget.23517 (PMC5788663; doi:10.18632/oncotarget.23517)
Supplement: Supplementary file 1 [file oncotarget-09-2603-s001.pdf]

Treatment-associated *TP53* DNA-binding domain missense mutations in the pathogenesis of secondary gliosarcoma

SUPPLEMENTARY MATERIALS

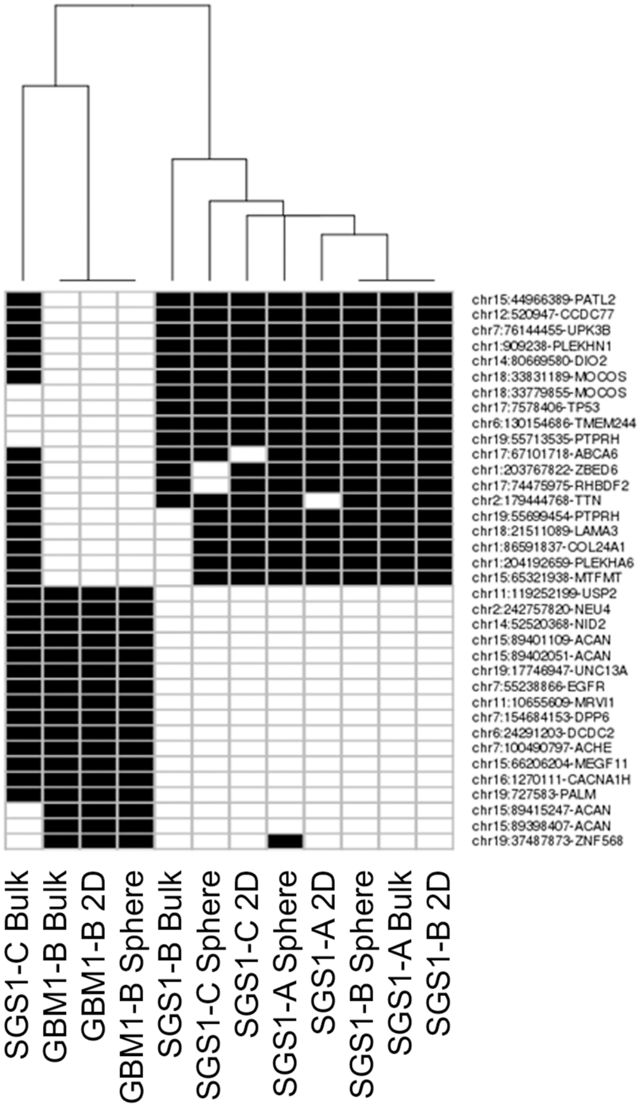

Supplementary Figure 1: Unsupervised hierarchical clustering analysis of GBM1 and SGS1 samples based on differentially expressed variants detected on RNA sequencing.

Supplementary Table 1: GBM1 and SGS1 distinguishing somatic SNVs identified by RNA sequencing

Sample Cluster	Gene	hg19 Coord	Base Change	Amino Acid Change	Functional Impact Score
SGS1	TP53	17:7578406	C>T	R175H	2.585
SGS1	MOCOS	18:33831189	C>A	H703N	2.56
SGS1	CCDC77	12:520947	A>C	S25R	2.38
SGS1	ZBED6	1:203767822	T>G	L391R	1.59
SGS1	MTFMT	15:65321938	A>G	V5A	1.39
SGS1	TP53	17:7579359	G>A	R110C	1.39
SGS1	LAMA3	18:21511089	A>G	S2834G	1.285
SGS1	PLEKHA6	1:204192659	G>A	A1029V	0.345
SGS1	RHBDF2	17:74475975	C>A	A67S	0
SGS1	UPK3B	7:76144455	A>C	R284R	0
SGS1	TTN	2:179444768	C>G	A20775P	-0.145
SGS1	MOCOS	18:33779855	C>T	T170I	-0.32
SGS1	PTPRH	19:55713535	G>A	H348Y	-0.46
SGS1	ABCA6	17:67101718	C>T	M875I	-0.55
SGS1	DIO2	14:80669580	T>C	T92A	-0.69
SGS1	COL24A1	1:86591837	G>A	A61V	-0.83
SGS1	TMEM244	6:130154686	A>G	F80L	-1.245
SGS1	PLEKHN1	1:909238	G>C	R539P	-1.32
SGS1	PATL2	15:44966389	T>C	M88V	-1.825
GBM1	PALM	19:727583	G>A	R53H	2.89
GBM1	MEGF11	15:66206204	G>A	L861F	2.14
GBM1	ACAN	15:89398407	C>T	P863L	2.14
GBM1	CACNA1H	16:1270111	G>A	R2060H	1.155
GBM1	NID2	14:52520368	C>T	G453D	0.55
GBM1	ACAN	15:89402051	A>G	I1964V	0.425
GBM1	ACAN	15:89401109	A>G	V1650V	0
GBM1	NEU4	2:242757820	G>A	G301R	-0.3
GBM1	ZNF568	19:37487873	C>T	A427V	-0.525
GBM1	MRVI1	11:10655609	C>T	A70T	-0.55
GBM1	ACAN	15:89415247	C>G	D2258E	-0.565
GBM1	DCDC2	6:24291203	T>C	S221G	-0.65
GBM1	ACHE	7:100490797	G>T	H353N	-0.705
GBM1	DPP6	7:154684153	T>C	L854P	-0.88
GBM1	UNC13A	19:17746947	A>G	L1034P	-2.135
GBM1	EGFR	7:55238866	A>G	noncoding	
